# A First Genome Survey and Genomic SSR Marker Analysis of *Trematomus loennbergii* Regan, 1913

**DOI:** 10.3390/ani11113186

**Published:** 2021-11-08

**Authors:** Eunkyung Choi, Sun Hee Kim, Seung Jae Lee, Euna Jo, Jinmu Kim, Jeong-Hoon Kim, Steven J. Parker, Young-Min Chi, Hyun Park

**Affiliations:** 1 Division of Biotechnology, College of Life Sciences and Biotechnology, Korea University, Seoul 02841, Korea; amy_choi@korea.ac.kr (E.C.); choihjdy@gmail.com (S.H.K.); skullcap@korea.ac.kr (S.J.L.); eunajo@kopri.re.kr (E.J.); rlawlsan04@korea.ac.kr (J.K.); 2 Greenwitch Co., 20, Jeungpyeong 2 Sandan-ro, Doan-myeon, Jeungpyeong-gun 27902, Korea; 3 Korea Polar Research Institute (KOPRI), Yeonsu-gu, Incheon 21990, Korea; jhkim94@kopri.re.kr; 4 National Institute of Water & Atmospheric Research Ltd. (NIWA), 217 Akersten Street Port Nelson, Nelson 7001, New Zealand; Steve.Parker@niwa.co.nz

**Keywords:** *Trematomus loennbergii*, scaly rockcod, repeat motif, SSR, microsatellite, Illumina

## Abstract

**Simple Summary:**

The scaly rockcod (*Trematomus loennbergii*) is distributed in the Antarctic Ocean and this area is isolated by the Antarctic Circumpolar Current. It is important region to study evolutionary diversity. Trematomus is the main genus, having 11 species, and their habit distribution is well known. However, their genetic and genomic information is not studied. In addition, some species have similar morphology. In this study, a genome survey of *T. loennbergii* and microsatellite motif analysis were conducted to obtain genomic profile. The fundamental data such as genome size, heterozygosity ratio, duplication ration and microsatellite motifs were obtained. These data will provide a foundation for further whole-genome sequencing and the development of new molecular markers of *T. loennbergii*.

**Abstract:**

*Trematomus loennbergii* Regan, 1913, is an evolutionarily important marine fish species distributed in the Antarctic Ocean. However, its genome has not been studied to date. In the present study, whole genome sequencing was performed using next-generation sequencing (NGS) technology to characterize its genome and develop genomic microsatellite markers. The 25-mer frequency distribution was estimated to be the best, and the genome size was predicted to be 815,042,992 bp. The heterozygosity, average rate of read duplication, and sequencing error rates were 0.536%, 0.724%, and 0.292%, respectively. These data were used to analyze microsatellite markers, and a total of 2,264,647 repeat motifs were identified. The most frequent repeat motif was di-nucleotide with 87.00% frequency, followed by tri-nucleotide (10.45%), tetra-nucleotide (1.94%), penta-nucleotide (0.34%), and hexa-nucleotide (0.27%). The AC repeat motif was the most abundant motif among di-nucleotides and among all repeat motifs. Among microsatellite markers, 181 markers were selected and PCR technology was used to validate several markers. A total of 15 markers produced only one band. In summary, these results provide a good basis for further studies, including evolutionary biology studies and population genetics of Antarctic fish species.

## 1. Introduction

The Antarctic shelf is the deepest ice shelf in the world. The depth of the Ross Ice Shelf, which is the largest ice shelf of Antarctica, is approximately 500 m, which is much deeper than the average depth (130 m) of other continental shelves. Moreover, the Southern Ocean is isolated by the Antarctic Circumpolar Current (ACC), and the temperature of the coastal water is cold with parts of it being frozen, making it an extreme environment. Therefore, the Antarctic Ocean is an important region for biologists to study survival adaptations and evolutionary diversification [1,2]. In the order Perciformes, Notothenioidei is the dominant suborder of fishes in the Antarctic area according to diversity and biomass [3], and fish species belonging to the family Notothenioidei have a variety of characters distinguishing them from other teleost fishes, such as the lack of hemoglobin and AFGP (antifreeze glycoprotein), which protect their body fluids from freezing [4,5]. The subfamily Trematominae is fundamental to the study of the coastal Antarctic ecosystem and includes only 14 species. *Trematomus* is the main most well-known genus in this family; it comprises 11 species, and their plasticity and diversity in habit distribution are well-known [6]. However, it is difficult to clearly distinguish between several *Trematomus* species because they have very similar morphologies. For example, *Trematomus loennbergii* and *Trematomus lepidorhinus* differ only in the absence or presence of scales on the preorbital and lower jaw [7]. *Trematomus loennbergii* Regan, 1913, known as the scaly rockcod, and the average length of this species is 20 cm, according to FishBase [8]. It is widely distributed in the Southern Ocean and is commonly found at a depth of over 300 m [9]. Its swimming activity is more spontaneous than that of other benthic fishes from the same family. The diet of females and males is similar; they feed on a wide range of prey, and their main food resources are epifaunal and tube-dwelling polychaetes [10].

*T. loennbergii* is an important species for studying evolution in the Antarctic area. Some morphological studies have emphasized the difficulty of distinguishing between this species and similar species. Owing to the development of molecular biology research techniques, especially next-generation sequencing (NGS) technology, genomic data, such as whole genome sequencing data, could be used for this purpose. Recently, some Antarctic species have been studied using this technology, but the genome of *T. loennbergii* has not been assessed to date. Therefore, low-coverage genome sequencing (e.g., genome surveys by K-mer analysis) needs to be carried out before performing large-scale sequencing to provide a genomic reference. Moreover, microsatellites or simple sequence repeats (SSRs), which consist of one to 10 nucleotides, are widely distributed throughout the genome of eukaryotes [11,12,13]. Therefore, in the present study, we conducted K-mer and QDD analyses to investigate the genome size and repeat sequences of *T. loennbergii* and to develop new microsatellite markers. These data can provide useful basic information on *T. loennbergii* genome.

## 2. Materials and Methods

### 2.1. Sample Collection and DNA Extraction

**Figure 1:**
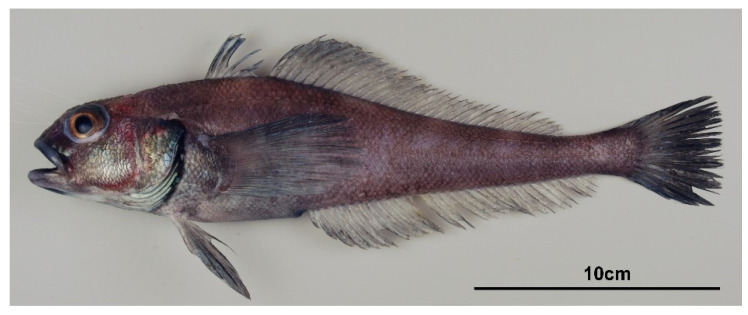
*T. loennbergii* from the Ross Sea, Antarctica.

A *T. loennbergii* (Figure 1) was caught in the Ross Sea (77°05′ S, 170°30′ E on CCAMLR Subarea 88.1), Antarctica, and stored in a freezer. The conventional phenol-chloroform method [14] was used for DNA extraction from the muscle tissue of frozen specimens. Quality check was conducted using a fragment analyzer (Agilent Technologies, Palo Alto, CA, USA), and DNA quantity was estimated using a Qubit 2.0 Fluorometer (Life Technologies, Carlsbad, CA, USA).

### 2.2. Library Construction and Sequencing

DNA library preparation was performed according to the Illumina Truseq DNA PCR-Free Library prep protocol. For library sample preparation, 2 μg of genomic DNA for 550 bp insert size was randomly sheared to yield DNA fragments using the Covaris S2 system (Covaris, Woburn, MA, USA). The fragments were blunt-ended, and a single ‘A’ nucleotide was added to the 3′ ends of the fragments for adaptor ligation. After the ligation step with adaptors having different sequences at the 5′ and 3′ ends of each fragment, library quality check was conducted using Bioanalyzer (Agilent Technologies, Santa Clara, CA, USA). The library was clustered on the Illumina cBOT station, and paired ends were sequenced for 101 cycles on an Illumina Novaseq 6000 sequencer (Illumina, San Diego, CA, USA) according to the Illumina cluster and sequencing protocols.

### 2.3. K-mer Analysis, Genome Assembly, and Microsatellite Analysis

After evaluation of quality values, all clean reads were used for K-mer analysis using Jellyfish and GenomeScope [15,16]. After estimating genome size, the assembly of *T. loennbergii* genome was conducted by MaSuRCA [17]. QDD version 3.1.2 pipeline [18] was used to identify the microsatellite motifs of *T. loennbergii*. The microsatellites repeat units in the genome were analyzed to calculate their length, quantity, and sequence. The following parameters were analyzed: number of mono-nucleotide repeats, di-nucleotides repeats, tri-nucleotide repeats, tetra-nucleotide repeats, penta-nucleotide repeats, and hexa-nucleotide repeat. These repeats were extracted from steps 1, 2 and 3. The parameter of each step was -contig 1, -make_cons 0 and -contig 1, respectively. After QDD analysis, total 181 primer set were selected by following parameters: motifs with more than five repetitions, 100–300 bp amplification product, 18–28 mer primer size and 58–62 °C for melting temperature. Among these primer pairs, 40 sets were randomly selected by 20 bp primer size and 60 °C annealing temperature. The PCR was conducted in total 20 μL including 5 μL genomic DNA (30 ng/μL), 10 μL 2× EmeraldAmp PCR Master Mix (Takara Bio, Shiga, Japan), 1 μL (10 pmole/L) each forward and reverse primers and 3 μL ddH_2_O. The PCR program was 2 min at 94 °C, followed by 35 cycles of 94 °C for 30 s, 60 °C for 30 s and 70 °C for 1 min, and the final extension was 10 min at 72 °C. The amplified PCR products were separately by 4% agarose gel electrophoresis and the 20 bp DNA ladder (Takara Bio, Shiga, Japan) was used to estimate the PCR product size.

## 3. Results and Discussion

### 3.1. Sequencing Data Statistics

In this study, the paired-end method with the Illumina NovaSeq platform was used to generate raw sequence data, and low-quality reads were filtered out. As a result, a total of 53.48 Gb of data were obtained. The data showed that Q20 was 96.3% and Q30 was 91.3% (Table 1). The Illumina NGS platform specifies that high-quality reads should have a Q20 of at least 90% and a Q30 of at least 85% [19]. Therefore, *T. loennbergii* sequencing data were highly accurate. Genome assembly and thereby the genome sequencing quality can be influenced by GC content; high GC content can reduce the sequencing coverage and cause sequencing bias. However, GC content between 30% and 50% has no effect on genome sequence quality [20,21,22]. In the present study, the GC content of *T. loennbergii* was 41.3% (Table 1); thus, it had no effect on the assembly results.

### 3.2. K-mer Analysis and Genome Size Prediction

**Figure 2:**
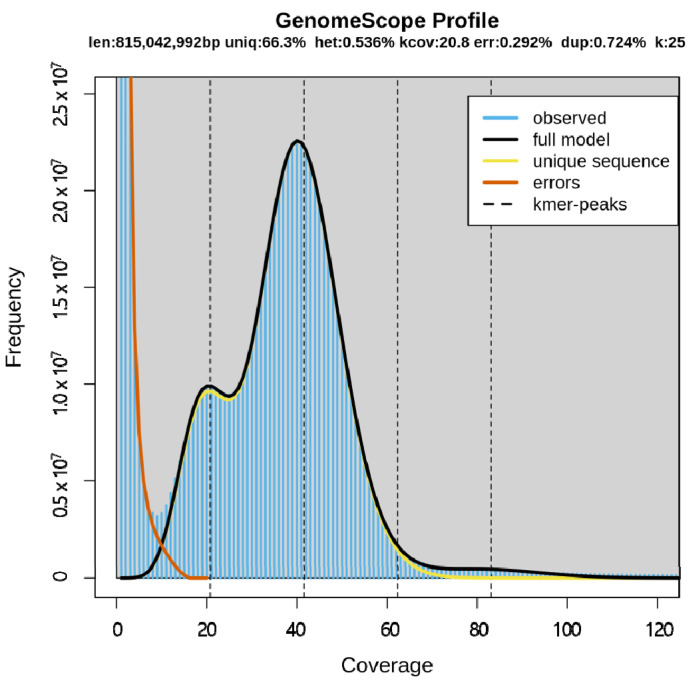
K-mer analysis (K = 25) of *T. loennbergii*. The *X*-axis represents coverage, and the *Y*-axis represents the frequency at each depth. This profile plot shows the fit of the model (black) to the observed frequency (blue).

Using the sequencing data, K-mer analysis was conducted and the 25-mer frequency distribution was the best. The estimated genome size was 815,042,992 bp (Table 2) and it is quite similar to the size of *Pogonophryne albipinna* (~883.8 Mb) which inhabits the deep waters of the Antarctic Southern Ocean [23] but smaller than the Antarctic blackfin icefish *Chaenocephalus aceratus (1.06 Gb)* [24]. The genome size of most fish is approximately 1 Gb, except for a few species [25], and the estimated genome size of *T. loennbergii* was similar to that of most fish species. Based on K-mer analysis, the heterozygosity was 0.536% and the average rate of read duplication was 0.724%. The sequencing error rate was 0.292%, and the highest frequency was near 40× coverage (Figure 2).

### 3.3. De Novo Assembly

To perform de novo assembly, MaSuRCA [17] was used with all clean reads. The total size of contigs was 820,644,295 bp, and the number of contigs was 613,288. The largest contig size was 59,484 bp, and the number of contigs larger than 1 K bp was 192,849 (31.4%). N50 contig length was 148,364 bp and L50 contig number was 1526. GC content was 40.65% and contig having more than 1K nt was 31.4% (Table 3). If heterozygosity rate is lower than 0.5%, it is not difficult to assemble [15], and heterozygosity rate of *T.* *loennbergii* is ~0.5%. In addition, high quality reads should have at least 85% on Q30 [19] and the high quality reads of *T.* *loennbergii* were 91.3%.

The assembly data is a first genome survey of *T. loennbergii* and it would be useful information for genomic research for *Trematomus* group. However, to improve whole genome sequencing and *de novo* assembly data, further study is needed to combine with more advanced technologies such as PacBio long read sequencing and high-throughput chromosome conformation capture (Hi-C).

### 3.4. Identification of Microsatellite Motifs

**Figure 3:**
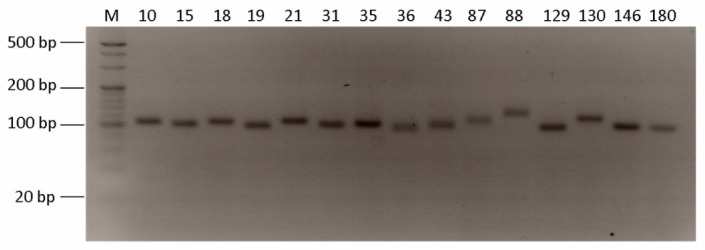
PCR products of microsatellite in *T. loennbergii*. M is the 20 bp DNA marker and 4% agarose gel electrophoresis was used. The number is primer pairs order.

Based on the genome assembly, QDD pipeline was used to identified SSR markers and the total number of identified microsatellite motifs was 2,264,647. Among them, di-nucleotide repeats were the most abundant (1,970,270, 87.00%) followed by tri-nucleotide repeats (236,541, 10.45%), tetra-nucleotide repeats (43,907, 1.94%), penta-nucleotide repeats (977,333, 0.34%), and hexa-nucleotide repeats (6196, 0.27%). The tendency of di-nucleotide repeats frequency in the studied species was similar to that in other fish species, such as *Pseudosciaena crocea* and *Megalobrama amblycephala* [26,27]. Furthermore, this result was consistent with data indicating that the repeat frequency decreases with the increase in repeat length because long mutations are related to high mutation rates [28]. The most frequent repeat motif among the four types of di-nucleotide repeats was the AC/GT repeat, and it was also the most abundant repeat motif (1,394,543, 61.57%) among all repeat motifs. The hexa-nucleotide repeat motif was the least frequent but had the highest number (n = 80) of repeat motif types (Table 4). After this analysis, we selected 181 microsatellite primer pairs (Table S1). Among these primer sets, we randomly chose 40 primer pairs and conducted PCR amplification with T. loennbergii. As a result, 15 primer pairs produced one clear band (Figure 3). Therefore, the present data applied for molecular genetic marker and further validation studies using various Trematomus groups are needed.

## 4. Conclusions

In the present study, a genome survey was conducted and the estimated genome size of *T. loennbergii* was reported. Furthermore, microsatellite analysis was performed to study this genome. We found that the genome size was approximately 815.04 Mb, heterozygosity rate was 0.536%, and GC content was 40.65%. In addition, microsatellite motifs analysis showed that the most abundant repeat motifs were di-nucleotide motif and the most abundant repeat type was the AC repeat motif, accounting for 61.57% of the total number of repeats. These findings will be helpful for future studies on population genetics and evolutionary biology of *T. loennbergii* and related species.

## Figures and Tables

**Table 1:** Sequencing data for the genome of *T. loennbergii*.

Raw Data (bp)	Q20 (%)	Q30 (%)	GC Content (%)
53,486,656,166	96.3	91.3	41.3

**Table 2:** Statistics of the estimated genome size by GenomeScope.

	17-mer	19-mer	25-mer
Genome size (bp)	774,371,521	786,515,471	815,042,992
Heterozygosity (%)	0.485	0.532	0.536
Duplication ratio (%)	0.798	0.744	0.724

**Table 3:** Statistics of *T. loennbergii* assembled genome sequences.

	MaSuRCA
Number of contigs	613,288
Total size of contigs	820,644,295
Longest contig	59,484
Number of contigs > 1000 nt	192,849 (31.4%)
Number of contigs > 10,000 nt	7790 (1.3%)
N50 contig length	148,364
L50 contig count	1526
GC content (%)	40.65

**Table 4:** Distribution of microsatellite motifs in *T. loennbergii*.

Repeat Motif	Number of Repeats								Total
	5	6	7	8	9	10	11–20	>21	
Di-nucleotide (1,970,270)									
AC/GT	562,651	252,660	135,367	89,123	61,221	45,302	170,290	77,929	1,394,543
AG/CT	188,161	64,236	32,609	16,413	9982	6453	21,335	5587	344,776
AT/AT	109,496	45,063	25,231	14,169	8234	6053	20,675	1362	230,283
CG/CG	521	104	22	21					668
Tri-nucleotide (236,541)									
AAT/ATT	29,084	12,994	7386	4347	2774	2076	3986	131	62,778
AGG/CCT	24,138	11,861	7937	5006	3504	1903	4472	212	59,033
AGC/GCT	14,871	6253	3373	1734	917	444	1211	81	28,884
AAC/GTT	13,646	6495	4094	2152	720	469	650	9	28,235
AAG/CTT	11,036	5730	2754	1535	815	616	1108	282	23,876
ATC/GAT	8035	3684	2516	1456	954	415	1576	360	18,996
ACC/GGT	3656	1960	1036	511	145	96	75		7479
ACT/AGT	2218	822	403	292	145	134	519	172	4705
CCG/CGG	1099	228	92	54			53		1526
ACG/CGT	643	248	87	15	6	30			1029
Tetra-nucleotide (43,907)									
ACAG/CTGT	1581	958	627	702	732	458	1192	21	6271
AGAT/ATCT	1360	940	606	399	366	319	1397	297	5684
ACGC/GCGT	1249	1089	656	364	279	175	263		4075
AGGG/CCCT	2503	1045	243	113	22		30		3956
AAAC/GTTT	1755	830	359	181	73	78	89		3365
ATCC/GGAT	945	676	357	51	195	120	502	15	2861
AATC/GATT	1121	616	370	124	100	75	68		2474
AAAG/CTTT	1214	317	162	175	76	72	117		2133
AAAT/ATTT	1508	301	97	24	6	24	53		2013
ACTC/GAGT	399	303	325	95	125	243	349	80	1919
ACAT/ATGT	583	314	62	178	117	27	339	33	1653
AAGG/CCTT	761	317	186	27	15	60	200	15	1581
Others (17)	2934	1297	548	387	205	90	420	41	5922
Penta-nucleotide (7733)									
AGAGG/CCTCT	481	275	250	83	109	156	756		2110
AAGAT/ATCTT	397	228	36	29			23		713
AAGGC/GCCTT	253	79	48	12		35	42		469
AATGT/ACATT	339	87			12				438
AAGCT/AGCTT	180	132	39				27		378
AGCAT/ATGCT	92	96	15	42	30		21		296
AAATC/GATTT	234	30	15						279
AAAGG/CCTTT	117	18	29		1		54		219
Others (49)	1795	421	214	122	121	60	89		2831
Hexa-nucleotide (6196)									
AACCCT/AGGGTT	664	468	202	128	75	57	105		1699
ACACGC/GCGTGT	377	167	171	102	176	114	12		1119
ACACTC/GAGTGT	78	72	85	187			96		518
ACACAT/ATGTGT	18	24	37	54	70	80	43		326
AAGAGG/CCTCTT	125	49							174
AATCAG/CTGATT	102	45							147
ACTCTG/CAGAGT	86	23	18			18			145
Others (73)	1092	416	299	70	60	43	88		2068
Total	993,598	423,971	228,963	140,477	92,382	66,295	232,325	86,636	2,264,647

## Data Availability

Data from the *T. loennbergii* genome project were deposited at NCBI under BioProject number PRJNA610666. The whole-genome sequence was deposited in the Sequence Read Archive (SRA) database under accession number SRX6919726.

## Supplementary Materials

The following are available online at https://www.mdpi.com/article/10.3390/ani11113186/s1, Table S1: List of microsatellite primers of *T. loennbergii.*

## References

[1] Lautrédou A.-C., Hinsinger D., Gallut C., Cheng C.-H., Berkani M., Ozouf-Costaz C., Cruaud C., Lecointre G., Dettai A. (2012). Phylogenetic footprints of an Antarctic radiation: The Trematominae (Notothenioidei, Teleostei). Mol. Phylogenet. Evol..

[2] Lannoo M.J., Eastman J.T. (2000). Nervous and sensory system correlates of an epibenthic evolutionary radiation in Antarctic notothenioid fishes, genus Trematomus (Perciformes; Nototheniidae). J. Morphol..

[3] Near T.J., Pesavento J.J., Cheng C.-H.C. (2004). Phylogenetic investigations of Antarctic notothenioid fishes (Perciformes: Notothenioidei) using complete gene sequences of the mitochondrial encoded 16S rRNA. Mol. Phylogenet. Evol..

[4] Clarke A., Johnston I.A. (1996). Evolution and adaptive radiation of Antarctic fishes. Trends Ecol. Evol..

[5] DeVries A.L., Cheng C.H.C. (2005). Antifreeze proteins and organismal freezing avoidance in polar fishes. Fish Physiol..

[6] Lautredou A.-C., Bonillo C., Denys G., Cruaud C., Ozouf-Costaz C., Lecointre G., Dettai A. (2010). Molecular taxonomy and identification within the Antarctic genus Trematomus (Notothenioidei, Teleostei): How valuable is barcoding with COI?. Polar Sci..

[7] DeWitt H.H., Heemstra P.C., Gon O. (1993). Nototheniidae In Fishes of the Southern Ocean.

[8] Fishbase. https://www.fishbase.in/summary/7057.

[9] Vacchi M., Greco S., La Mesa M. (1991). Ichthyological survey by fixed gears in Terra Nova Bay (Antarctica). Fish list and first results. Mem. Biol. Mar. Oceanogr..

[10] La Mesa M., Vacchi M., Castelli A., Diviacco G. (1997). Feeding ecology of two nototheniid fishes, Trematomus hansoni and Trematomus loennbergii, from Terra Nova Bay, Ross Sea. Polar Biol..

[11] Gemayel R., Cho J., Boeynaems S., Verstrepen K.J. (2012). Beyond junk-variable tandem repeats as facilitators of rapid evolution of regulatory and coding sequences. Genes.

[12] Pérez-Jiménez M., Besnard G., Dorado G., Hernandez P. (2013). Varietal tracing of virgin olive oils based on plastid DNA variation profiling. PLoS ONE.

[13] Phumichai C., Phumichai T., Wongkaew A. (2015). Novel chloroplast microsatellite (cpSSR) markers for genetic diversity assessment of cultivated and wild Hevea rubber. Plant Mol. Biol. Rep..

[14] Sambrook J., Russell D.W. (2006). Purification of nucleic acids by extraction with phenol: Chloroform. Cold Spring Harb. Protoc..

[15] Marçais G., Kingsford C. (2011). A fast, lock-free approach for efficient parallel counting of occurrences of k-mers. Bioinformatics.

[16] Vurture G.W., Sedlazeck F.J., Nattestad M., Underwood C.J., Fang H., Gurtowski J., Schatz M.C. (2017). GenomeScope: Fast reference-free genome profiling from short reads. Bioinformatics.

[17] Zimin A.V., Marçais G., Puiu D., Roberts M., Salzberg S.L., Yorke J.A. (2013). The MaSuRCA genome assembler. Bioinformatics.

[18] Meglécz E., Pech N., Gilles A., Dubut V., Hingamp P., Trilles A., Grenier R., Martin J.F. (2014). QDD version 3.1: A user-friendly computer program for microsatellite selection and primer design revisited: Experimental validation of variables determining genotyping success rate. Mol. Ecol. Resour..

[19] Li G.-Q., Song L.-X., Jin C.-Q., Li M., Gong S.-P., Wang Y.-F. (2019). Genome survey and SSR analysis of Apocynum venetum. Biosci. Rep..

[20] Cheung M.-S., Down T.A., Latorre I., Ahringer J. (2011). Systematic bias in high-throughput sequencing data and its correction by BEADS. Nucleic Acids Res..

[21] Zhou W., Hu Y., Sui Z., Fu F., Wang J., Chang L., Guo W., Li B. (2013). Genome survey sequencing and genetic background characterization of Gracilariopsis lemaneiformis (Rhodophyta) based on next-generation sequencing. PLoS ONE.

[22] Shangguan L., Han J., Kayesh E., Sun X., Zhang C., Pervaiz T., Wen X., Fang J. (2013). Evaluation of genome sequencing quality in selected plant species using expressed sequence tags. PLoS ONE.

[23] Jo E., Cho Y.H., Lee S.J., Choi E., Kim J., Kim J.-H., Chi Y.M., Park H. (2021). Genome survey and microsatellite motif identification of Poonophryne albipinna. Biosci. Rep..

[24] Kim B.-M., Amores A., Kang S., Ahn D.-H., Kim J.-H., Kim I.-C., Lee J.H., Lee S.G., Lee H., Lee J. (2019). Antarctic blackfin icefish genome reveals adaptations to extreme environments. Nat. Ecol. Evol..

[25] Chen S., Xu W., Liu Y. (2019). Fish genomic research: Decade review and prospect. J. Fish. China.

[26] Li Q., Li Z., Dai G., Cao Y., Chen X., Chen L., Shangguan J., Ning Y. (2014). Isolation and characterization of eleven microsatellite loci in the marbled rockfish, Sebastiscus marmoratus (Scorpaenidae). Conserv. Genet. Resour..

[27] Zeng C., Gao Z., Luo W., Liu X., Wang W., Zhang X. (2013). Characteristics of microsatellites in blunt snout bream (Mega27. lobrama amblycephala) EST sequences using 454 FLX. Acta Hydrobiol. Sin..

[28] Katti M.V., Ranjekar P.K., Gupta V.S. (2001). Differential Distribution of Simple Sequence Repeats in Eukaryotic Genome Sequences. Mol. Biol. Evol..

